# The Pivotal Role of Regulatory T Cells in the Regulation of Innate Immune Cells

**DOI:** 10.3389/fimmu.2019.00680

**Published:** 2019-04-09

**Authors:** Emeka B. Okeke, Jude E. Uzonna

**Affiliations:** ^1^Department of Pharmaceutical Sciences, University of Michigan, Ann Arbor, MI, United States; ^2^Department of Immunology, Faculty of Medicine, University of Manitoba, Winnipeg, MB, Canada

**Keywords:** cytokines, dendritic cells, neutrophils, inflammation, lymphocyte, homeostasis, immune tolerance, monocytes/macrophages

## Abstract

The distinction between innate and adaptive immunity is one of the basic tenets of immunology. The co-operation between these two arms of the immune system is a major determinant of the resistance or susceptibility of the host following pathogen invasion. Hence, this interactive co-operation between cells of the innate and adaptive immunity is of significant interest to immunologists. The sub-population of CD4^+^ T cells with regulatory phenotype (regulatory T cells; Tregs), which constitute a part of the adaptive immune system, have been widely implicated in the regulation of the immune system and maintenance of immune homeostasis. In the last two decades, there has been an explosion in research describing the role of Tregs and their relevance in several immunopathologies ranging from inflammation to cancer. The majority of these studies focus on the role of Tregs on the cells of the adaptive immune system. Recently, there is significant interest in the role of Tregs on cells of the innate immune system. In this review, we examine the literature on the role of Tregs in immunology. Specifically, we focus on the emerging knowledge of Treg interaction with dendritic cells, macrophages, neutrophils, and γδ T cells. We highlight this interaction as an important link between innate and adaptive immune systems which also indicate the far-reaching role of Tregs in the regulation of immune responses and maintenance of self-tolerance and immune homeostasis.

## Introduction

The immune system protects the host against pathogen invasion and is therefore armed with an arsenal of deadly ammunition (cells and proteins) necessary for the elimination of microbes or substances determined to pose significant threat to the normal functioning of the host. This inherent function made it imperative that the evolution of host immunity encompasses important mechanisms to prohibit the destruction of self. Hence, the maintenance of immune tolerance is central to the normal functioning of the immune system and breakdown of immune tolerance results in catastrophic consequences to the host.

Increase in knowledge of the immune system has greatly helped to delineate the important mechanisms involved in maintenance of immune tolerance. It is now known that a core aspect of lymphocyte development is the elimination of lymphocytes that are reactive to self-ligands by the process of negative selection (1). It seems pertinent that the immune system develop fail-safe mechanisms to handle self-reactive lymphocytes that were not eliminated during the process of negative selection. One such mechanism that has been proposed for decades is the ability of a subtype of T lymphocytes to suppress the function of other lymphocytes. This sub-population of T lymphocytes identified by the expression of CD4, CD25, and FOXP3 has been shown to be a major player in the maintenance of immune tolerance and homeostasis (2, 3). These naturally occurring regulatory T cells (Tregs) comprise 5–10% of peripheral CD4^+^ T cells in the circulation, have potent suppressive abilities and were initially known to suppress CD4^+^CD25^−^ T cells (4). Recently, Tregs have been implicated in the regulation of other cells of the adaptive immune system including CD8^+^ T cells and B cells (5–7).

It is well-known that the co-operative interaction between cells of the innate immune system and cells of the adaptive immune system is crucial for the induction of adequate immune response. Recent findings that Tregs regulate the function of cells of the innate immune system like macrophages, dendritic cells, and neutrophils are intriguing and indicate the significant overlap between both arms of immunity. In this review, we discuss the essential role of Tregs (defined by the expression of CD4, CD25, and FOXP3) in the maintenance of immune tolerance. In particular, we focus on the emerging role of Tregs as regulators of cells of the innate immune system. We highlight the under-explored interaction between Tregs and cells of the innate immune system and the significance of this interaction in the maintenance of immune tolerance and in the pathogenesis of autoimmune diseases.

## Regulatory T Cells and Immunity

Several lines of evidence summarized in Table 1, led to the development of Treg biology. The history of Tregs, the biological origin and the terms of classification of Treg subtypes have been well-reviewed elsewhere (3, 29, 30) and highlighted for the reader's information.

**Table 1:** Summary of findings leading to discovery of Tregs.

**Year**	**Discovery**	**References**
1970, 1972	Ability of thymocytes to induce lymphocyte Suppression was reported.	(8, 9)
1972, 1973	Introduction of the concept of suppressor T cells (Tsups).	(10–12)
1976	Identification of the phenotype of Tsups based on cell surface antigens.	(13, 14)
1976	Report that the I-J region of the MHC is responsible for Tsups activity.	(15, 16)
1983, 1986	RNA and DNA screening fails to identify the I-J region of the MHC responsible for Tsups activity leading to the demise of Tsups.	(17, 18)
1995	Identification of CD4^+^ CD25^+^ T cells as regulatory T cells (Tregs).	(19, 20)
1997	Identification of T regulatory type 1 (Tr1) cells.	(21)
1998	CD4^+^CD25^+^ Tregs shown to be a distinct lineage of suppressor cells.	(22, 23)
2001	Identification of Tregs function as the cause of Scurfy and IPEX syndromes in mice and humans, respectively.	(24, 25)
2003	Identification of the transcription factor forkhead box P3 (FOXP3) as essential for Treg function.	(26–28)

It is worthy of note that in addition to the naturally occurring Treg cells, CD4^+^ T cells with regulatory phenotype can be induced *in vivo* and *in vitro* with antigenic stimulation in the presence of IL-10. These so called IL-10-producing T regulatory type 1 (Tr1) cells (31) usually do not express FOXP3 and have been shown to have potent suppressive ability (21, 32). Notably, Tr1 cells are able to inhibit CD4^+^ T cell responses through IL-10 dependent and independent mechanisms (33–37). Importantly, Tr1 cells are distinct from FOXP3^+^ Tregs (natural Tregs) because they do not constitutively express FOXP3. Also, Tr1 cells have been shown to function separately from FOXP3^+^ Tregs in certain conditions (38, 39). The biology and functional characteristics of Tr1 cells have been recently reviewed exhaustively (40, 41) and these articles are recommended for readers wanting more information on these cells.

Tregs were originally identified as a subset of immune cells critical for the maintenance of self-tolerance and prevention of autoimmune diseases (19). However, since their discovery, Tregs have been ascribed the eminent role of an omnipotent wonder regulatory cell that is paramount in nearly all immunological responses such as oral tolerance (42), fetal-maternal tolerance (43), infectious tolerance (44), transplantation tolerance (45), allergen-induced hypersensitivities (46), and even immune memory (47).

In their landmark paper, Sakaguchi et al. initially showed that Tregs protect the host from autoimmune diseases (19). They showed that transfer of CD4^+^ cells depleted of CD25^+^ population into athymic syngeneic nude mice resulted in autoimmune pathologies in several organs. Additionally, they demonstrated the significant role of Tregs in maintenance of transplantation tolerance by showing that depletion of Tregs leads to heightened rejection of allogeneic skin grafts (19). Since then, several studies have associated defective Treg function with the development of several autoimmune diseases. In mice, a mutation in the FOXP3 gene leads to a lethal wasting disease characterized by exaggerated CD4^+^ T cell activity (25). An analogous autoimmune disease in humans known as immune dysregulation, polyendocrinopathy, enteropathy X-linked (IPEX) syndrome is associated with the dysfunction of FOXP3 gene (24). In animal studies, depletion of Tregs leads to rapid and severe onset of arthritis and adoptive transfer of Tregs rescues the animals from the disease (48). In humans, reduced Treg populations are associated with the exacerbated form of juvenile idiopathic arthritis and rheumatoid arthritis (49, 50). Similarly, a mutation in FOXP3 gene is associated with spontaneous development of inflammatory bowel disease (IBD) (26) and a phase 1 clinical trial of Treg therapy in patients with refractory Crohn's disease was found to be effective (51). Also defective Treg function has been implicated in the development of type 1 diabetes (52), multiple sclerosis (53), and atopic dermatitis (54). Indeed, there is overwhelming experimental evidence of the significance of Tregs in the prevention of autoimmune diseases and the current challenge is the translation of this knowledge to effective clinical therapy for patients with autoimmune diseases.

The role of Tregs in maintenance of host immunity during infection is controversial. While some studies indicate that the suppressive nature of Tregs limit the immune response to infection and is detrimental to the host, other studies have shown that Tregs are essential for the successful elimination of pathogens and the prevention of pathogen-induced immunopathologies. For example, in the case of sepsis (systemic inflammatory response to infection), Venet et al. showed that increased numbers of Tregs is associated with poor outcome (55). In contrast, Heuer et al. reported that adoptive transfer of *in vitro* stimulated Tregs increased bacterial clearance and improved survival in murine model of sepsis (56). Also, Cambos et al. showed that Tregs suppress excessive inflammation in lethal *plasmodium chabaudi adami* infection in which mortality is associated with systemic inflammatory response (57). Tregs have also been shown to be protective in viral infections. Lund et al. demonstrated that Tregs facilitated the recruitment of immune cells for protection against herpes simplex virus in mice (58).

We recently showed that immunological or genetic inhibition of Tregs function by using an anti-CD25 monoclonal antibody (anti-CD25 mAb) treatment or mice lacking functional Tregs (CD25 KO mice), respectively, was detrimental in a sepsis model of bacterial infection or LPS-induced acute inflammatory response (59). This was associated with exaggerated production of pro-inflammatory cytokines including IL-1β, IL-6, IL-12, TNF, and CCL2. Strikingly, adoptive transfer of Tregs from wild-type mice into CD25 KO mice before LPS challenge rescues them from an otherwise acute death (59).

The initial work of Sakaguchi et al. indicated that Tregs regulate CD4^+^CD25^−^ Th cells since their depletion leads to exaggerated CD4^+^ Th cell response resulting in immunopathology (19). This role of Tregs in regulating CD4^+^ Th cell function has also been reported in several studies (52, 53, 60). We recently showed that Tregs regulate CD4^+^ Th cells in a murine model of sepsis (61). Following Treg depletion, CD4^+^ Th cells exhibit increased cellular activity in response to LPS which leads to exuberant activation of other immune cells such as macrophages resulting in excessive inflammatory response, organ damage, and mortality (61). In addition to regulation of CD4^+^ Th cells, Tregs have also been shown to regulate other cells of the adaptive immune system like CD8^+^ T cells and B cells (5, 6). However, a limited number of studies have examined the role of Tregs in the regulation of innate immune cells. We will focus on the essential interaction between Tregs and cells of the innate immune system in the maintenance of immune homeostasis in the remainder of this review.

## Tregs and Dendritic Cells

The role of a specialized group of immune cells, aptly called antigen presenting cells (APCs), in providing the necessary second signal for lymphocyte activation is highly appreciated. Dendritic cells (DCs) were first identified as Langerhans cells in the skin in 1868 but their primary role in immune response was not recognized until the 1970s (62). Although their primary role in immune response was only recently discovered, DCs are the most potent APCs *in vitro* and *in vivo* (63). Currently, DCs are appreciated as the sentinels of the immune system. They are present in tissues and in the peripheral circulation, surveying the host immune system for the presence of antigens and upon antigen encounter rapidly upregulate co-stimulatory molecules and migrate to the lymph node where they present antigens to T cells (63). Additionally, DCs secrete cytokines like IL-12 that can activate T cells. The role of DCs in the immune response has been well-reviewed (63–65), hence we will focus on the crosstalk between DCs and Tregs in the regulation of the immune response.

Paradoxically, DCs that are essential for the activation of the immune response have also been implicated in the induction of immune tolerance. Finkelman et al. showed that DCs can induce both immune activation and immune tolerance. They showed that in the absence of additional stimuli, injection of mice with a rat IgG2b anti-DC mAb leads to T cell specific tolerance to rat IgG (66). Also, Hawiger et al. showed that targeted delivery of antigen via DC-restricted endocytic receptor, DEC-205, leads to T cell unresponsiveness or anergy (67). However, in the presence of a second signal such as may be provided by co-injection of anti-CD40 agonistic antibody, immune activation is observed instead of tolerance (67). Hence, DCs can tilt the immune response to tolerance or activation if insufficient signal is received. This is understandable as unwarranted immune activation can result in autoimmune pathologies. It is thought that the induction of tolerance by DCs is dependent on the degree of DC maturation. Immature or semi-mature DCs that encounter antigen that does not result in full maturation induce immune tolerance while full antigenic maturation of DCs leads to immune activation (68).

One way by which DCs help in the maintenance of immune tolerance is by induction of Tregs. Compared to B cells and macrophages, DCs are more efficient in the induction of Tregs (69) and DC-depleted APCs show a significant reduction in the ability to induce Tregs (70). Studies have shown that, double positive thymocytes are selected for commitment to Treg lineage depending on the intensity of their response to self-antigens presented by thymic DCs (71). It appears that the underlying factor which determines Treg induction by DCs is the strength of the antigenic stimuli. For example, Kretschmer et al. demonstrated that subimmunogenic antigen delivery to DCs resulted in the generation of Tregs from naïve CD4^+^ T cells in the periphery (72). This observation is supported by other reports which show that strong activation of DCs leads to production of cytokines which inhibit Treg induction (73, 74). Thus, it appears that there is an immunogenic threshold observed by DCs below which DCs help to maintain immune tolerance through the induction of Tregs.

**Figure 1:**
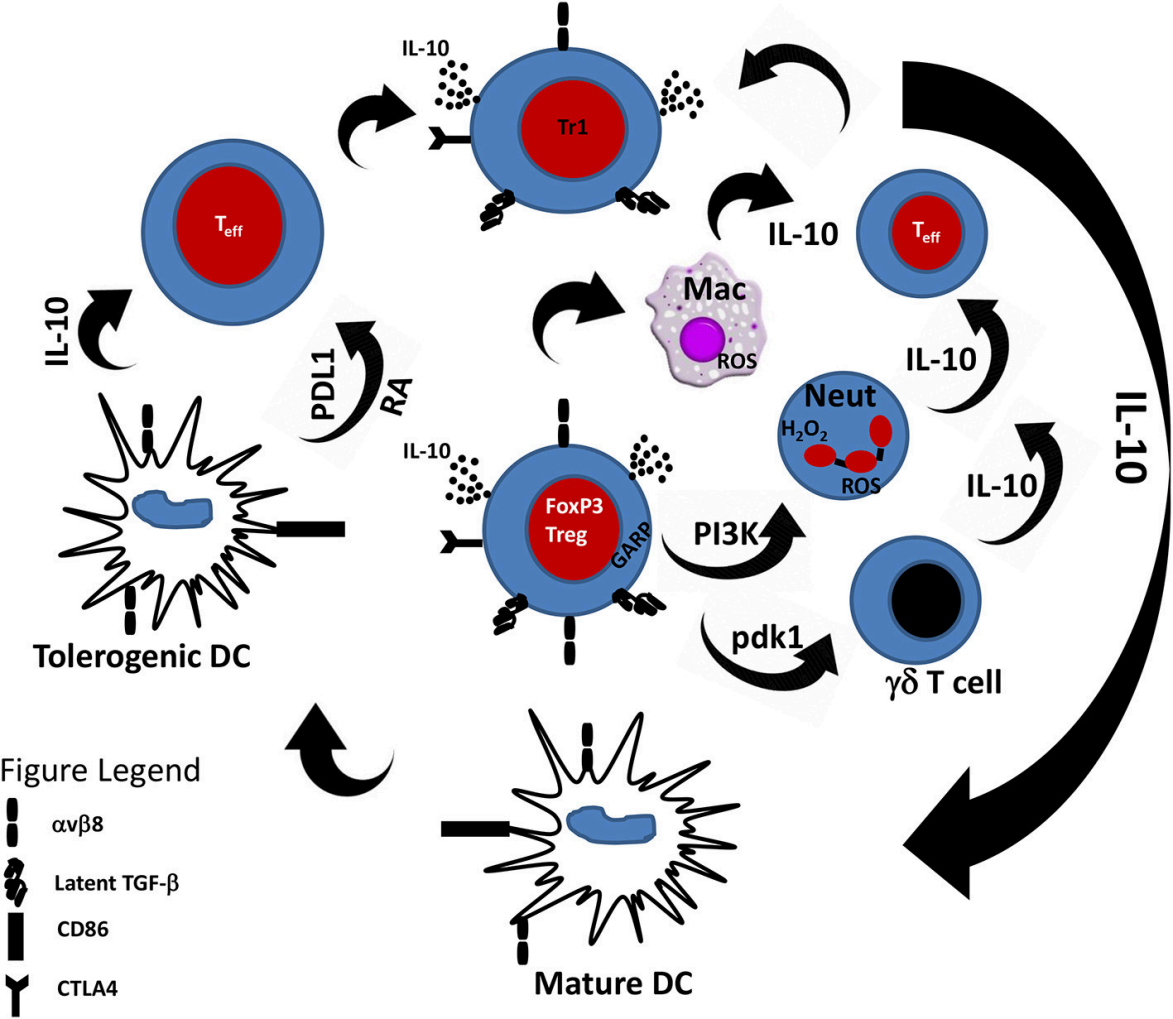
Cross-talk between Tregs and innate immune cells. Tregs through the secretion of IL-10 and TGF-β are able to modulate the response of innate immune cells toward an anti-inflammatory phenotype. Likewise, innate immune cells in the presence of IL-10 can induce Tr1 cells that can suppress effector T cell response. PDL1, Programmed death-ligand 1; RA, Retinoic acid.

How does DCs promote Treg induction? Studies have shown that DCs promote Treg induction through cytokine and non-cytokine mediators. For example, the induction of Tregs by DCs has been shown to be mediated through the inhibitory molecule programmed death-ligand 1 (PD-L1) expressed on DCs. Hence, genetic or immunological inhibition of PD-L1 on DCs leads to their inability to induce FOXP3^+^ Tregs even in the presence of the necessary cytokine signals (75). Treg expansion and differentiation by DCs is highly favored in the presence of certain cytokines. In particular, the cytokines IL-10 and TGF-β are important in the induction of Tregs by DCs. The regulatory function of IL-10 has been well-studied in the context of several immunopathologies. IL-10 is a potent inhibitor of the immune response (76) and deficiency of IL-10 has been shown to result in exaggerated immune responses to pathogens leading to immunopathology (77–79). Indeed, it is well-established that IL-10 induces the class of Tr1 cells which are functionally similar to naturally occurring Tregs (32). Importantly, Tr1 cells have been shown to mediate their regulatory function through the production of IL-10 (38, 80, 81). *In vitro*, activation of effector T cells (Teff) with IL-10-treated DCs leads to anergy and generation of Tr1 cells (31, 82, 83). IL-10-treated DCs have potent suppressive ability and have been shown to be effective in experimental treatment of allergic asthma (84), graft-vs.-host disease (85) and inflammatory bowel disease (86). In addition to induction of Tr1 cells through exogenous IL-10, DCs also induce Tr1 cells by the secretion of IL-10 (87, 88). Hence, there is a positive feedback mechanism by which IL-10 induces tolerogenic DCs which then acquire the ability to secrete IL-10 and in turn induce tolerance through induction of Tr1 cells. Since Tregs produce IL-10, it follows that Tregs can also induce tolerogenic DCs and this two-way relationship between Tregs and DCs via IL-10 is a critical mechanism of maintaining immune tolerance (Figure 1).

In addition to mediating tolerance through IL-10, DCs have also been shown to mediate tolerance through the immunoregulatory cytokine TGF-β. The essential role of TGF-β in the maintenance of immune tolerance has been well-established. TGF-β is important for the development and function of FOXP3^+^ T cells (89, 90) and Tregs also mediate tolerance through the secretion of TGF-β (91). Mice lacking TGF-β signaling develop a fatal lymphoproliferative disease similar to scurfy mice (92, 93). Interestingly, DCs have been shown to induce FOXP3^+^ Tregs from FOXP3^−^ precursors in the presence of exogenous and endogenous TGF-β (70). Evidence of the role of DCs in the maintenance of immunological tolerance through TGF-β is compelling. Laouar et al. demonstrated that targeted functional inactivation of TGF-β receptor signaling in DCs resulted in enhanced T cell responses in experimental autoimmune encephalomyelitis (EAE) (94). Yamazaki et al. showed that CD8^+^CD205^+^ DCs induce Tregs via the production of TGF-β (95). In line with this, antibody neutralization of TGF-β abrogates the ability of CD8^+^CD205^+^ DCs to induce Tregs (95). Travis et al. showed that the induction of Tregs by DCs via TGF-β is mediated by the cytokine activating α_v_β_8_ integrin on DCs (96). They showed that targeted disruption of α_v_β_8_ on DCs leads to autoimmune disease. In contrast disruption of α_v_β_8_ on T cells does not lead to autoimmune disease indicating that TGF-β signaling through DCs is paramount for the maintenance of immune tolerance. Furthermore, mice with DCs lacking α_v_β_8_ have reduced Tregs in the colon. Additionally, DCs lacking α_v_β_8_ lose the ability to induce Tregs *in vitro* (96). On the other hand, Worthington et al. showed that Tregs also express high amounts of αvβ8, which enables them to activate latent TGF-β for the suppression of T cell-mediated inflammation (97). In line with this, recent studies show that Tregs mediate their suppressive function by activating latent TGF-β1 presented by GARP (glycoprotein A repetitions predominant) to integrin αVβ8 on their surface (98–100). Collectively, these studies demonstrate the essential role of TGF-β1 in Treg function.

Studies have also shown that DCs produce the vitamin A metabolite—retinoic acid (RA) and that RA-producing DCs are important for Treg induction (101). Interestingly, Tregs can be generated *de novo* from peripheral T cells in human or murine blood by RA (102–104). Also incubation of Tregs with TGF-β and RA increases their suppressive ability (105). Additionally, intestinal DCs treated with an antagonist against RA receptor lose their ability to induce Tregs (105). The mechanism by which RA leads to the induction of tolerogenic DCs is not completely understood. Studies have shown that RA-producing enzyme retinal dehydrogenase 2 (RALDH2), which is encoded by *Aldh1a2* gene, is highly expressed by DCs in mesenteric lymph node (MLN) and peyer's patches compared to DCs from other lymphoid organs (101, 106). Recent work by Ohoka et al. showed that RA and granulocyte-macrophage colony-stimulating factor (GM-CSF) induced the expression of *Aldh1a2* in DCs through the interaction of RA receptor and retinoid X receptor complex and subsequent activation of the transcription factor sp1 (107). Indeed, accumulating evidence suggests that RA-producing DCs play an essential role in the maintenance of oral tolerance (108). T or B cells activated in the presence of RA are “imprinted” to express gut-homing receptors (106). Also, Siewert et al. showed that Tregs preferentially migrate to the gut following treatment with RA (109). It has been proposed that RA produced by DCs increase TGF-β-dependent induction of Tregs from naïve T cells by inhibiting their differentiation into inflammatory T cells. In line with this, Balmer and Blomhoff found that RA increases TGF-β receptor subunit signaling (110). The role of RA in the maintenance of oral tolerance is understandable since the gut is constantly barraged by exposure to foreign and commensal antigens and maintenance of tolerance without compromising immunity is a priority. Previous studies identified CD103^+^ DCs in the gut-associated lymphoid tissue (GALT) as the subset of DCs that produce RA necessary for Treg induction in the gut (105). However, recent findings indicate that RA production by DCs is not restricted to the CD103^+^ DCs in the GALT. Guilliams et al. found CD103^−^ RA-producing DCs in the skin and lung draining lymph nodes (111). These observations highlight the essential role of different subsets of DCs in the maintenance of immune tolerance via production of RA.

## Tregs and Macrophages

Macrophages have long been appreciated as important immune cells that help to maintain immune homeostasis via phagocytosis of foreign matter, apoptotic or necrotic cells (112). As an important part of the innate immune system, they possess several pathogen recognition receptors (PRRs) and are quickly mobilized to the infection site preceded only by neutrophils. Macrophages play an important role in shaping the adaptive immune response. For example, macrophages present antigens to T and B cells and also secrete several cytokines which directs the responses of T and B cells (113).

It is now well-known that macrophages assume distinct phenotype and function based on their microenvironment. Hence, macrophages are broadly classified into two—the proinflammatory M1 macrophages which arise in the presence of cytokines like IFN-γ and IL-12, and the anti-inflammatory M2 macrophages (114). M2 macrophages are further subdivided based on the cytokine signals that give rise to them: M2a (IL-4 or IL-13), M2b (IL-1β, LPS, and immune complexes), and M2c (IL-10, TGF-β, or glucocorticoids) (115). Unlike the M1, M2 macrophages especially the M2c subset have a regulatory phenotype (116) and the regulatory role of macrophages have been of significant interest in recent years.

The relationship between Tregs and macrophages and the role of macrophages in the maintenance of immune tolerance has been severely under-explored compared to Treg/DC interaction. However, there is evidence that Treg/macrophage interaction significantly modulates host immune response. For example, co-culture of macrophages and Tregs leads to reduced expression of HLA-DR on macrophages and reduced production of proinflammatory cytokines in response to LPS stimulation (117). Recently, our group showed that depletion of Tregs leads to exaggerated macrophage activation that results in mortality to endotoxic shock (61).

Currently, there is significant interest in the role of regulatory macrophages (Mregs) in inflammation and cancer and the cross-talk between Tregs and Mregs. As described above, macrophages (M2c) assume a regulatory phenotype in the presence of TGF-β and IL-10, two cytokines that are widely associated with Treg function. This is in addition to the induction of anti-inflammatory phenotype in macrophages following treatment with glucocorticoids (118). Interestingly, the secretion of IL-10 is a characteristic feature of Mregs (119) and IL-10 plays a major role in the function of Mregs. For example, targeted disruption of IL-10 receptor signaling in macrophages leads to the spontaneous development of severe colitis (120). Since Tregs are also major producers of IL-10, it is conceivable that macrophage/Treg cross-talk via IL-10 plays a significant role in maintenance of tolerance. Indeed, Tregs are able to direct macrophage differentiation to the M2 regulatory phenotype in a mechanism that has been shown to be dependent on IL-10 and TGF-β (117, 121–123). Tregs have also been shown to induce macrophage regulatory phenotype through other mechanisms. Miwa et al showed that Tregs play a role in the maintenance of fetal-maternal tolerance through the upregulation of the enzyme indoleamine 2,3-dioxygenase (IDO) in monocytes (124). Venet et al. showed that Tregs play a major role in regulation of monocyte survival by promoting monocyte apoptosis in a mechanism dependent on the Fas/Fas ligand pathway (125).

These studies indicate that Mreg/Treg cross-talk is central to the maintenance of immune tolerance. Indeed, M2 macrophages have also been shown to induce Tregs (126, 127) and similar to Tregs, Mregs have shown therapeutic efficacy in the experimental treatment of inflammatory diseases like allergy and type 1 diabetes (128, 129) highlighting the role of Mregs in immune tolerance. In addition to the induction of Tregs by IL-10 and TGF-β activity, the ability of Mregs to induce Tregs has been shown to involve the production of reactive oxygen species (ROS). Disruption of the NAPDH-oxidase complex which is involved in ROS production impairs induction of Tregs by macrophages (130). Also, mice with reduced ROS production ability is more susceptible to autoimmune diseases compared to their wild type litter mates highlighting the role of macrophage ROS in immune tolerance (131). Hence, Mreg/Treg cross-talk is a major mechanism of immune tolerance that needs to be further explored.

## Tregs and Neutrophils

Neutrophils are one of the first responder cells of the innate immune system during bacterial infection and inflammation and constitute a hallmark of innate immunity (132, 133). They are the most abundant type of leukocytes in humans and express all known TLRs except TLR3. Hence, neutrophils are critical for the activation of innate immune defenses and defective neutrophil function leads to increased susceptibility to infections (134). The role of neutrophils in shaping the innate and adaptive immune responses has been extensively reviewed (135, 136). Here, we will focus on Treg/neutrophil interaction and the regulatory role of neutrophils in immunity.

There is accumulating evidence that neutrophils can be polarized to attain unique phenotypes in response to the microenvironment. For example, Fridlender et al. described the characterization of anti-tumorigenic neutrophil (N1 phenotype) and pro-tumorigenic neutrophil (N2 phenotype) in mice (137). The presence of TGF-β in the tumor microenvironment induces N2 neutrophils while blockade of TGF-β favors the N1 phenotype. There is also evidence that neutrophils which are renowned for their pro-inflammatory activity can exhibit regulatory and immuno-suppressive functions through various mechanisms. For example, Schmielau et al. demonstrated that neutrophil-derived hydrogen peroxide suppresses T cell responses in patients with cancer (138). This finding was confirmed by the work of Pillay et al. which showed that neutrophil-derived hydrogen peroxide suppresses T cell responses in sepsis through the expression of Mac-1 (139). These immuno-suppressive neutrophils were characterized as CD16^+^CD54^high^ cells in contrast to the classical CD16^+^CD54^lo^ neutrophils (139). The suppressive function of neutrophils has also been demonstrated in viral infections. Bowers et al. demonstrated that neutrophils purified from the blood of HIV-1-infected patients suppress T cell function through PD-L1/PD-1 interaction and production of ROS (140).

In addition to ROS production, studies have shown that the regulatory function of neutrophils can be associated with cytokine production. Again, the cytokine IL-10 has been implicated in this regard. Doz et al. demonstrated that IL-10-producing neutrophils inhibit Th17 cell responses during mycobacterial infection (141). This was supported by the work of Zhang et al. which showed that co-activation of Syk kinase and MyD88 adaptor protein pathways leads to IL-10 production by murine neutrophils which dampen immune response in mycobacterial infection (142).

There is evidence of cross-talk between Tregs and neutrophils and there is significant interest in the role of Treg/neutrophil interaction in the maintenance of immune homeostasis. The work of Himmel et al. showed that Tregs are able to induce neutrophil recruitment through the production of CXCL8 (143). It is also conceivable that IL-10 production by Tregs can modulate neutrophil function and vice versa. Lewkowicz et al. showed that activated Tregs upregulate the expression of suppressor of cytokine signaling 3 (SOCS 3) in neutrophils and induce IL-10 and TGF-β production (144).

Overall, it is important to note that the relationship between Tregs and neutrophils has been severely underexplored. An important area of interest is Treg/neutrophil interaction in inflammatory and autoimmune diseases. The pro-inflammatory function of neutrophils in the promotion and pathogenesis of several autoimmune diseases like vasculitis (145), rheumatoid arthritis (146), and systemic lupus erythematosus (147) has been well-described. Interestingly, defective Treg function has been demonstrated in all these diseases and Treg therapy has proven to be useful in their management (148). Therefore, one can speculate that defective Treg function is associated with exaggerated neutrophil activity. Indeed, we and others have found this to be the case. Richards et al. showed that Tregs limit inflammation in the skin by inhibiting neutrophil accumulation and survival (149). We recently showed that reduced Treg numbers in mice leads to exaggerated neutrophil activity resulting in mortality in endotoxic shock (150). We also found that Tregs regulate survival and activity of human and murine neutrophils and co-culture of Tregs and neutrophils increases neutrophil apoptosis (150). This is in line with the work of Lewkowicz et al. which showed that LPS-activated Tregs inhibit neutrophil function and promote their apoptosis (151). More work is required to delineate the role of Treg/neutrophil interaction in autoimmune and inflammatory diseases and this remains an active area of investigation in our laboratory.

## Tregs and Gamma Delta (γδ) T Cells

Majority of T cells develop in the thymus, have T cell receptor (TCR) composed of αβ chains, and are mostly found in peripheral lymphoid organs. In contrast, there are a sub-population of T cells that develop within and outside the thymus, have TCR composed of γδ chains and are very few in peripheral lymphoid organs but abundant in intra-epithelial compartments. This population of T cells are called γδ T cells (152). γδ T cells are considered innate immune cells due to their innate-like characteristics. Notably, unlike conventional T cells, they can be activated without the help of APCs and do not require MHC class I or II peptide presentation (153). Also, similar to innate immune cells, γδ T cells have been shown to carry out phagocytosis (154). The biology and role of γδ T cells in immunity has been comprehensively reviewed by others (155–157) and the reader is referred to these excellent reviews for detailed information on these aspects of γδ T cells.

There is evidence that γδ T cells possess both inflammatory and regulatory properties. Proinflammatory γδ T cells are classified according to their production of either IFN-γ or IL-17 (158–160) and have been implicated in the pathogenesis of several autoimmune diseases (161) including EAE (162, 163) and collagen-induced arthritis (164). In contrast, stimulation of γδ T cells in the presence of TGF-β leads to the induction of FOXP3 expressing γδ T cells with suppressive phenotype (165, 166). These appropriately called regulatory γδ T cells have been shown to exert their suppressive function through the production of IL-10 and TGF-β to inhibit T cell activation and proliferation (165–168).

Few studies have examined the bilateral relationship between Tregs and γδ T cells and there is evidence of cross-talk between these two populations. Since, γδ T cells with regulatory phenotype are induced in the presence of IL-10 and TGF-β and Tregs secrete these cytokines, it follows that Tregs can induce γδ T cells with regulatory phenotype. Indeed, this has been demonstrated recently. Park et al. showed that Tregs maintain intestinal homeostasis by suppressing γδ T cells (169). They showed that CD4^+^ T cell specific deletion of the phosphoinositide dependent protein kinase 1 (*Pdk1*) gene leads to defective Treg function and the constitutive activation of colitis-inducing γδ T cells which is inhibited by adoptive transfer of wild-type Tregs (169). The findings of Park et al. was confirmed by Yurchenko et al. who unequivocally demonstrated the role of Tregs in regulating pathogenic γδ T cells in the intestines (170).

Other studies have also demonstrated the ability of Tregs to regulate the function of γδ T cells. Li et al. showed that Tregs inhibited cytokine production by γδ T cells in response to *M tuberculosis* antigen (171). Xu et al. showed that in pediatric epilepsy associated with inflammation of the central nervous system, there is increase in the number of γδ T cells and this corresponds to a decrease in Tregs numbers in the epileptogenic lesions (172). Additionally, they demonstrated that seizures were significantly decreased following inhibition of γδ T cells activity or adoptive transfer of Tregs into these mice. Overall, these findings highlight the ability of Tregs to regulate the function of another innate immune cell—γδ T cells.

## Concluding Remarks

There is unequivocal experimental evidence of the role of Tregs in the maintenance of immune tolerance (20). Initial studies focused on the role of Tregs in the regulation of CD4^+^ Th cells (52, 53, 60). However, as the knowledge of Tregs biology increased, they were shown to be able to regulate other cells of the adaptive immune system including CD8^+^ T cells and B cells (5, 6). In this review, we highlighted the burgeoning function of Tregs in the regulation of cells of the innate immune system including dendritic cells, macrophages, neutrophils, and γδ T cells. In addition to these cell types, Tregs have also been shown to regulate the function of other innate immune cells including natural killer (NK) (173) and innate lymphoid (ILC) cells (174). Indeed, as shown in Figure 1, this cross-talk between Tregs and innate immune cells is important for maintenance of immune tolerance and regulating the pathogenesis of inflammatory diseases. Hence, Tregs appear to be the master-regulatory cell type necessary for immune tolerance involving both the innate and adaptive immunity (175).

Previous studies have highlighted the role of Tregs in regulating pathogenic CD4^+^ T cells (52, 53, 60). In this review, we emphasize the significance of Tregs in regulating innate immune cells and that defective Treg function is associated with aggravated inflammatory response by innate immune cells. We postulate that defective Treg function simultaneously affects adaptive immune cells and innate immune cells in different disease models. For example, we previously showed that depletion of Tregs leads to exaggerated CD4^+^ T cell response in bacteria and LPS-induced acute inflammation models (61). In another study, we demonstrated that reduced Treg numbers also leads to enhanced neutrophil activity in LPS-induced inflammation (150). Hence, it follows that reduced Treg numbers simultaneously affects CD4^+^ T cells and neutrophils. It is worthy of note that defective Treg function can lead to exaggerated activity of adaptive immune cells like CD4^+^ T cells, which in turn will lead to the aggravated immune response of innate immune cells like macrophages (61). Likewise, defective Treg function can also lead to exaggerated activity of innate immune cells whose cytokine secretion will aggravate the immune response of adaptive immune cells. This highlights the master regulatory role of Tregs in the maintenance of immune homeostasis.

A possible explanation of the simultaneous effect of Treg function on innate and adaptive immune cells is the ability of Treg cells to extract ligands from APCs by trogocytosis (176). For example, previous studies have shown that Tregs exert their suppression through the inhibitory receptor CTLA-4 (177, 178). It is worthy of note that CTLA-4 shares its ligands (CD80 and CD86) with the co-stimulatory receptor CD28. Interestingly, Qureshi et al. showed that Tregs suppress T cell response by using CTLA-4 to extract CD80 or CD86 from APCs leading to impaired co-stimulation of T cells through CD28 (179). This implies that decrease in number of Tregs relative to APCs will limit trogocytosis of CD80 and CD86 by CTLA-4^+^ Tregs leading to higher CD28 costimulation and more aggressive T cell response (180). Another study showed that Tregs that acquired CD86 from DCs have enhanced ability to suppress T cell responses (176). Overall, these studies indicate that co-operation between Tregs and APCs shapes immune homeostasis.

The regulation of innate immunity by Tregs is not surprising. The immune system is made up of a complex network of cells that are intricately involved in the immune network. Hence, as shown in Figure 1, it is understandable that IL-10-producing innate immune cells induce Tregs and vice versa. Indeed, cellular cytokine activity is a proven way of recruiting responder cells for immune function. Delineating the complex interaction among different cell types is a daunting task and remains the subject of investigation in several laboratories around the world. Although the distinction between innate and adaptive immunity is one of the basic tenets of immunology, there is general agreement that the co-operation between these two arms of the immune system is a major determinant of resistance or susceptibility of the host to pathogen invasion. Recently, accumulating evidence suggests that there is a fine line separating the two arms of the immune system. As highlighted in this review, Treg/DC, Treg/macrophage Treg/neutrophil, and Treg/γδ T cell cross-talks are critical for the maintenance of immune tolerance and prevention of autoimmune diseases. Thus, the immune system is a highly integrated network and although stratifying immune function can be beneficial, immunity is better understood in the context of cooperative functionality of cells.

## Author Contributions

EO did all literature search required for the review and wrote the first draft of the manuscript. JU edited and corrected the manuscript draft for publication.

### Conflict of Interest Statement

The authors declare that the research was conducted in the absence of any commercial or financial relationships that could be construed as a potential conflict of interest.
